# Functional Characterization of Pepper Vein Banding Virus-Encoded Proteins and Their Interactions: Implications in *Potyvirus* Infection

**DOI:** 10.3390/v12091037

**Published:** 2020-09-17

**Authors:** Pallavi Sabharwal, Handanahal S. Savithri

**Affiliations:** Department of Biochemistry, Biological Sciences Building, Indian Institute of Science, Bangalore 560012, Karnataka, India; pallavi.sbl@gmail.com

**Keywords:** Pepper vein banding virus, Potyvirus, virus-like particles, intrinsically disordered proteins, protease, B-domain, plant nanoparticles

## Abstract

Pepper vein banding virus (PVBV) is a distinct species in the *Potyvirus* genus which infects economically important plants in several parts of India. Like other potyviruses, PVBV encodes multifunctional proteins, with several interaction partners, having implications at different stages of the potyviral infection. In this review, we summarize the functional characterization of different PVBV-encoded proteins with an emphasis on their interaction partners governing the multifunctionality of potyviral proteins. Intrinsically disordered domains/regions of these proteins play an important role in their interactions with other proteins. Deciphering the function of PVBV-encoded proteins and their interactions with cognitive partners will help in understanding the putative mechanisms by which the potyviral proteins are regulated at different stages of the viral life-cycle. This review also discusses PVBV virus-like particles (VLPs) and their potential applications in nanotechnology. Further, virus-like nanoparticle-cell interactions and intracellular fate of PVBV VLPs are also discussed.

## 1. Introduction

The genus *Potyvirus*, named after its type species, *Potato virus Y* (PVY) [1], in the family Potyviridae is the largest group of plant RNA viruses that are also economically very important [2]. Potyviruses cause significant loss to agricultural productivity by infecting economically important crops—especially those belonging to the Cucurbitaceae, Solanaceae, Cruciferae and Compositae family [3,4]. Apart from infecting agriculture crops, potyviruses have wild plant hosts as well [5,6].

**Figure 1:**
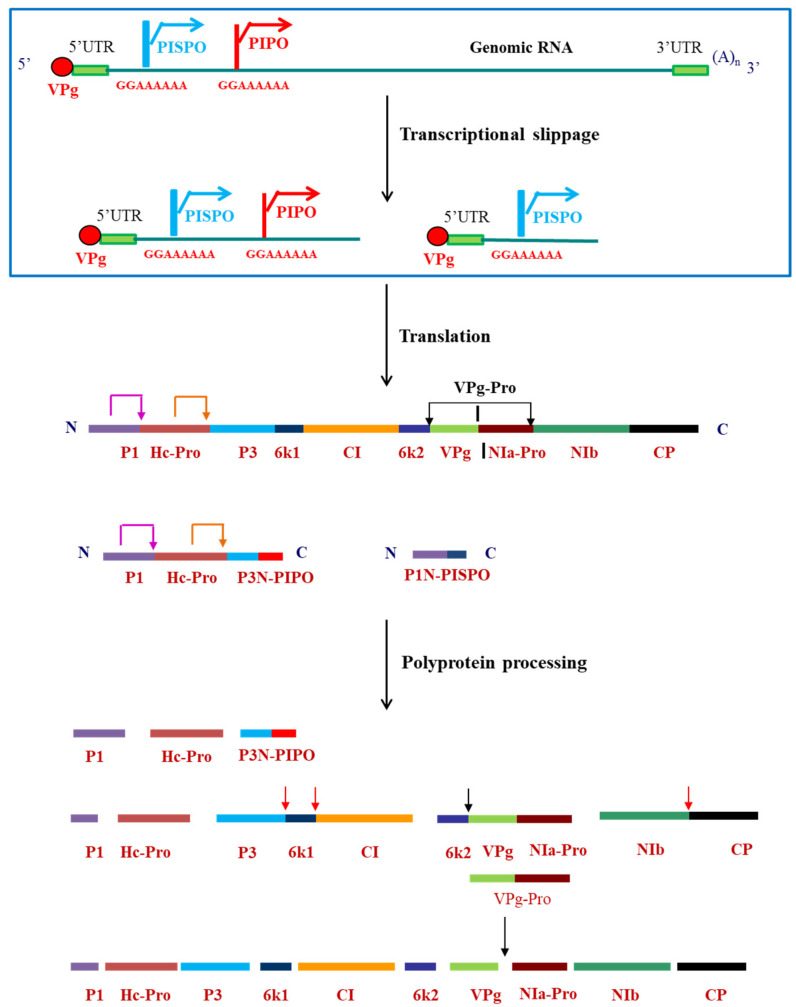
Genome organization of viruses belonging to *Potyvirus* genus, indicating the transcriptional slippage at the slippery sequences and polyprotein processing by the viral-encoded proteases to form mature proteins. Pink arrow at the P1 and orange arrow at the HC-Pro cleavage sites indicate self-cleavage of the two proteases. Red arrows indicate *trans* cleavage by VPg-Pro and black arrows indicate the *cis* cleavage by VPg-Pro.

In the Indian subcontinent, pepper/chili is an economically important crop. However, a major bottleneck in its cultivation was crop loss due to infection by viruses, particularly by pepper vein banding virus (PVBV) [3,4]. PVBV infects Solanaceae plants such as bell-pepper (*Capsicum annuum*) and chili (*Capsicum frutescens*) and thus pose a major threat to the agriculture. In bell-pepper, systemic infection of PVBV leads to development of symptoms like mottling, leaf malformation, elongation of petiole and narrowing of leaves [4]. Potyviruses are transmitted by aphids in a non-persistent and non-circulative manner [7] and by seeds to the progeny of infected plants. The aphid transmission takes place by interaction of the virions with aphids via helper component proteinase (HC-Pro) which is a multifunctional potyviral protein having various functional motifs [8]. As with other potyviruses, PVBV virions also bind to aphid mouthparts indirectly through interaction with HC-Pro. The genomic sequence of PVBV was the first potyviral sequence to be reported from India [9,10]. The 5′-UTR and 3′UTR are 163 and 281 nucleotides in length, respectively, with multiple CAA repeats present in the 5′UTR along with two conserved regions—potybox-a (AACACAACAU) and potybox-b (CUCAAGC) [9]. The 5′ end of the genomic RNA is covalently linked via a phosphodiester linkage to the viral protein genome linked (VPg) and the 3′ end is polyadenylated [9]. Like in other potyviral RNA genomes, PVBV genome encodes for a single ORF which is translated to generate a polyprotein of 3088 amino acids. This polyprotein is processed by the viral-encoded proteases, P1, HC-Pro and NIa-Pro into ten functional and mature proteins [10]. Within the P3 cistron of potyviruses, there is a coding sequence first identified in turnip mosaic virus (TuMV) known as PIPO (pretty interesting potyviridae ORF) [11] which is formed by transcriptional slippage at the conserved G_1-2_ A_6-7_ motif in the P3 nucleotide sequence. Another overlapping ORF was identified in the P1 cistron of sweet potato feathery mottle (SPFMV) group of potyvirus, known as PISPO (pretty interesting sweet potato potyvirus ORF) [12,13], also formed due to the transcriptional slippage at the G_1-2_ A_6-7_ slippery sequence in the P1 ORF. Both these ORFs were also located in the PVBV sequence. The genome organization of viruses belonging to *Potyvirus* genus along with the embedded ORFs and the slippery sequence is shown in Figure 1.

The proteins encoded by potyviruses are multifunctional, have multiple interacting partner proteins and also participate in various events of the viral life cycle. This functional flexibility could be due to the intrinsically disordered proteins (IDPs)/and intrinsically disordered protein regions (IDPRs) encoded by potyviruses. IDPs are the proteins with large number of distinct and dynamic conformations [14]. This property of intrinsic disorder was specifically studied with PVBV-encoded VPg in the current review. IDPs are natively unfolded proteins which are unfolded along their entire length whereas IDPRs have just a stretch of ≥30 disordered residues within a folded protein [15]. IDPs are involved in one to many and many to one interaction which thus allow the same protein to perform multiple functions. A large proportion of viral proteins exhibit partial or completely disordered structure [16]. The structural disorder in IDPs allow them to interact with their cognate partners with high specificity and low affinity [17]. IDPs undergo a transition to form a more rigid structure known as disorder-to-order transition by virtue of their interaction with specific cognate partner proteins [17,18,19,20]. The potyviral proteins such as VPg, CP and P1 have IDPRs which are also known to render multifunctionality to these proteins [21,22,23,24,25,26,27]. Both VPg and P1 contain molecular recognition features (MoRFs) in their N-terminal and C-terminal disordered region, respectively [23,28]. MoRFs are the short regions within the disordered region of a protein which are involved in protein-binding and also undergo a disorder-to-order transition upon binding with a cognate partner protein [29].

Here, we discuss the functional characterization of different proteins encoded by PVBV with an emphasis on their multifunctionality and regulation. The focus is on the PVBV-encoded proteins involved in the initial steps of the potyviral infection viz assembly/disassembly of virions, virus replication, translation and polyprotein processing. The detailed analysis of these steps by virtue of the proteins involved in them has provided some insights into the potyviral infection cycle. Characterization of the functions and interactions of the proteins encoded by PVBV may also shed light on the different mechanisms by which potyviral proteins participate in infection and are regulated by the host. Potyviruses are also being exploited in various biotechnological applications, therefore the functional characterization of virus-like particles (VLPs) formed by the heterologous expression of PVBV coat protein (CP) and their route of internalization into mammalian cells is discussed. The use of chimeric VLPs as a potential therapeutic agent has also been elaborated in the current review.

## 2. Disassembly and Reassembly of PVBV

**Figure 2:**
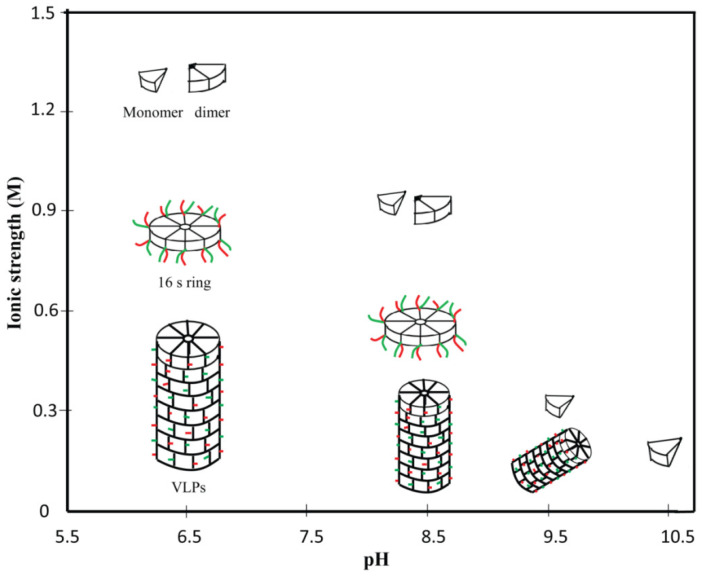
Effect of ionic strength and pH in the disassembly of pepper vein-banding virus (PVBV) virus-like particles (VLPs). At pH < 6.5 and a low ionic strength, VLPs are the dominant species which get dissociated into 16 S ring and further into coat protein (CP) subunits (monomer/dimer) as the ionic strength keeps on increasing. As the pH increases, even a low ionic strength is sufficient to disassemble the VLPs into ring intermediate. At higher ionic strength (>0.9 M) and high pH (>10), VLPs are completely dissociated into individual CP subunits.

Assembly and disassembly are two crucial steps in the life cycle of a virus. PVBV CP when expressed in *E. coli* was shown to form flexuous rods of varying length and encapsidated the CP mRNA [9] which formed the basis for studying the assembly of this flexuous rod-shaped virus. The assembly of flexuous rods require a large number of macromolecular interactions which still some time ago were poorly understood when compared to rigid rods like tobacco mosaic virus (TMV) [30]. Therefore, PVBV VLPs were used as a model to understand the assembly and disassembly of the potyvirus [31]. The monomeric CP subunits interact with each other in a head to tail manner to form dimers, which then form an octameric 16S ring-like intermediate. This ring-like intermediate is made up of 8 subunits, hence the diameter of potyviruses is ~10 nm [32]. The 16S rings ultimately assemble to form VLPs. The N-terminal 53 and C-terminal 23 residues have been demonstrated to be important for the initiation of assembly, as deletion of these residues resulted in assembly incompetent CP subunits. The N-terminal 53 and C-terminal 23 residues of PVBV consist of mostly charged residues, therefore electrostatic interactions between them are responsible for association of CP subunits. Removal of these residues either by deletion or trypsin digestion prevents formation of CP dimers. However, once the virions are formed these regions are exposed and are dispensable for infectivity of the virus and stability of the VLPs. VLPs are sensitive to the environment and can disassemble under conditions of high pH or salt concentration. The interplay of ionic strength vs. pH in the disassembly of VLPs is depicted in Figure 2.

Potyviral CP has been shown to undergo post-translational modifications such as phosphorylation and glycosylation. It was shown that phosphorylation of CP at the core region diminished its capacity to bind RNA, suggesting that phosphorylation could regulate the assembly/disassembly process [33,34]. It has been reported in many proteins that glycosylation reverses the effect of phosphorylation [35]. The *O*-glycosylation of the coat protein of plum pox virus (PPV) has been shown to enhance viral infection [36]. Thus, these closely related post-translational modifications could likely regulate the assembly/disassembly of potyviruses.

## 3. Polyprotein Processing: Multifunctional VPg-Pro–the Major Player

The polyprotein formed after translation undergoes proteolytic processing which is mediated by three viral-encoded proteases—P1, HC-Pro and NIa-Pro. While P1 and HC-Pro have *cis* cleavage activity and cleave themselves off from the C-terminus [37], the NIa-Pro has both *cis* and *trans* cleavage activities [38] and cleaves at seven sites within the polyprotein (Figure 1). NIa-Pro cleaves in *cis* (Figure 1; black arrows) at the junctions between CI/6 K2, 6 K2/VPg, VPg/NIa-Pro and NIa-Pro/NIb and in *trans* (Figure 1; red arrows) at the junctions between P3/6K1, 6K1/CI and NIb/CP [39,40,41,42,43]. The cleavage site between VPg and NIa-Pro is suboptimal [44] having glutamate rather than glutamine at the P1 position (P1 position in the recognition sequence). Thus, having an N-terminal VPg domain and C-terminal NIa-Pro domain, NIa-Pro exists mostly as VPg-Pro. Any mutation at this cleavage site, which either increases/decreases the rate of cleavage, renders the virus replication-defective [44,45].

NIa-Pro from PVBV was demonstrated to be a serine-like cysteine protease with Asp 81 and Cys 151 present at the active site [41]. These residues along with His 46 are conserved across potyvirids and organized as the catalytic triad responsible for the function [46,47]. NIa-Pro recognizes a heptapeptide sequence and is specific towards the cleavage at either Q/A, Q/S or Q/T, flanked by specific residues on either side of this site [48]. PVBV NIa-Pro shares 35%–50% amino acid sequence identity with 28 viral proteases deposited in the Swiss-Prot database. It has a sequence identity of 47% and 45% with tobacco etch virus (TEV) protease and tobacco vein mottling virus (TVMV) protease, respectively. The three dimensional structures of these two proteases have been determined earlier [49,50]. The modeled structure of PVBV NIa-Pro showed a two domain anti-parallel β barrel fold similar to those of chymotrypsin like proteases and a surface exposed loop encompassing Ser129 and Trp143 that was shown to be most flexible. However, mutation of Trp143 (to Ala143) of PVBV NIa-Pro resulted in almost complete loss of activity. MD (Molecular dynamics) simulation studies showed that, the mutation altered the orientation of Cys 151 such that it moved away from the other catalytic triad residues (His 46, Asp 81) and therefore the enzyme would be inactive [24]. Based on this, a W–C loop (W143-C151) was identified in PVBV NIa-Pro which contains the active site Cys151. This loop was shown earlier to be crucial for interaction with substrates and products [49,50]. The presence of VPg at the N-terminus of NIa-Pro was shown to enhance the protease activity of NIa-Pro when present in *cis*. Further, when the two domains are present in *trans*, there was a marked increase in the turnover number [24]. It was proposed that VPg binds to NIa-Pro around the region encompassing Ser 129 and Trp 143 and favorably orients the W–C loop residues as well as the catalytic Cys151 such that it leads to an increase in the catalytic efficiency and enzymatic activity of the protease.

The N-terminal VPg domain, apart from acting as an activator of the protease, serves as a primer during viral replication and as a hub of the interaction-network due to the presence of intrinsically disordered regions within the protein [51]. PVBV VPg harbors the Walker A and Walker B motifs at the N-terminal region which is typical of proteins involved in NTP-binding and hydrolysis. It was shown that although VPg does not have ATPase activity on its own, it gains this function upon interaction with NIa-Pro in *cis* or *trans* [52]. Lys 47 (PVBV numbering) of walker A motif was identified to be crucial for the ATPase function of PVBV VPg-Pro. Lys 47 of VPg has been an important residue for a number of potyviral functions viz uridylylation, suppression of RNA silencing, RNA-binding, interaction with host proteins, viral RNA stability and virus viability [25,53,54,55]. Therefore, ATPase activity of VPg-Pro manifested by virtue of Lys 47, could be utilized during VPg uridylylation, polyprotein processing and other processes at different stages of the potyviral life cycle [18].

The N-terminal disordered region of PVBV VPg was identified as the domain involved in interaction with NIa-Pro and dynamics of interaction between these domains was studied. For this purpose, N-terminal deletion mutants were generated to understand structure-function relationship of VPg-Pro by virtue of the intrinsically disordered region. It was shown that N-terminal disordered 22 residues of VPg were important for interaction with NIa-Pro and also essential for modulating the protease activity of NIa-Pro [28]. The deletion of 22 residues from the N-terminus of PVBV VPg resulted in the gain in the secondary and the tertiary structure of the protein. These structural changes brought about a gain in the ATPase function of ∆N22 VPg in the absence of its cognate-binding protein, NIa-pro. It was thus speculated that the N-terminal disordered region of PVBV VPg could be essential for modulating the structure and function of VPg and NIa-Pro domains [28]. The N-terminal residues of VPg in different potyviruses also mark the domain of interaction with various other partner proteins of both host and viral origin [56,57,58]. Therefore, the N-terminal 22 residues of PVBV VPg constitute the MoRFs which may be responsible for its moonlighting properties [28]. The MoRF residues have also been predicted in VPg from different potyviruses and are conserved across various potyvirus species [59]. Not only VPg from plant RNA viruses like potato virus A (PVA), lettuce mosaic virus (LMV), PVY, sesbania mosaic virus (SeMV) are known to be disordered [25,26,27,60], but also the poliovirus VPg and foot-and-mouth disease virus (FMDV) VPg are shown to be IDPs. While the NMR structure of poliovirus VPg has been determined, [61] potyviral VPg structure is not yet known. PVA VPg was found to be capable of forming a more compact structure in the presence of anionic phospholipids as well as SDS [27]. The picornaviral VPgs are composed of only 20–30 amino acids whereas the potyviral VPgs are of approximately 200 amino acids in length and hence the latter are more complex at the molecular level. The structural flexibility thus imparts functional diversity to VPg. This structural flexibility of VPg, therefore, could be explained by a more complex “one-gene–many-proteins–many-functions” model rather than the classical “one gene–one protein–one structure–one function” view [15].

## 4. Replication of PVBV—Highlighting the Multifunctional NIb

Replication of potyviruses involves synthesis of negative-sense RNA complementary to the genomic RNA, which is then used as a template for synthesizing positive-sense progeny RNA. This process requires viral polymerase, several other viral and host proteins as well as the host membrane and is thus mediated by various types of RNA–protein and protein–protein and protein–lipid interactions. Detailed mechanism of replication in potyviruses has not been elucidated but based on the similarity in the genome organization; it was proposed that replication strategy of potyviruses could be similar to the picornaviruses.

Among potyviral proteins, Nuclear Inclusion-b (NIb) protein has the RNA-dependent RNA polymerase (RdRp) activity [39]. Interactions of NIb with VPg-Pro (via either VPg or protease domain), and the CI protein have been shown to be critical for in vivo NIb polymerase activity [62,63,64,65]. NIb has two catalytic activities-uridylylation of VPg and RNA-dependent RNA polymerase activity. It has the canonical Gly-Asp-Asp (GDD) motif required for divalent metal ion-binding, which allows interaction with RNA. A direct evidence of polymerase activity of NIb was demonstrated for the first time when the recombinant TVMV NIb was shown to utilize cognate RNA as a template for RNA synthesis [66].

PVBV NIb when overexpressed in *E. coli* and purified under non-denaturing conditions could recognize poly (A) as the template and synthesize complimentary RNA only in the presence of oligo (dT) primer [67]. PVBV NIb harbors the conserved active site motif Gly-Asp-Asp or (GDD) which corresponds to residues 351–353. PVBV NIb could uridylylate VPg in a template independent manner. The N- and C-terminal deletion analysis of VPg revealed that N-terminal 21 and C-terminal 92 residues of PVBV VPg are dispensable for in vitro uridylylation [67]. Between the N-terminal 21 and C-terminal 92 residues of PVBV VPg, two conserved tyrosines were present—Tyr 42 and Tyr 66. Tyr 42 formed the nucleotide-binding motif, AYTTKKGK which in the case of PVA VPg was shown to be responsible for nucleotide-binding [68]. Upon deletion of this motif in PVBV VPg, the uridylylation reaction was debilitated, but not completely abrogated. However, when Tyr 66 was mutated to Thr 66, complete loss of uridylylation by PVBV NIb was observed. Tyr 66 is part of NMY motif, suggested to be involved in RNA attachment. Therefore, PVBV VPg could be linked to viral RNA via Tyr 66 [67]. In PVA, in the absence of Tyr at the corresponding position, Tyr119 has been suggested as an alternate site for uridylylation [53]. Thus, in potyviruses, replication could begin with uridylylation at a tyrosine residue of VPg which acts as primer for progeny RNA synthesis as observed in other picornaviruses and comoviruses. Although, in the case of the latter a serine residue is nucleotidylylated [68,69,70,71].

NIb undergoes post-translational modifications such as sumoylation, which causes the redistribution of NIb from nucleus to the cytoplasm, a key step for the viral replication. Thus, sumoylation of NIb is responsible for regulating the nucleo–cytoplasmic partitioning of NIb. The sumoylation of NIb could also play a crucial role in suppressing the host defense mechanism thereby promoting the potyvirus infection [72]. Besides this, potyviral NIb is also a target of autophagy where a key component of autophagy (Beclin1) binds to the GDD motif of NIb [73]. However, potyviruses could subvert and exploit the autophagy to promote/enhance their pathogenicity through VPg by mediating the degradation of SGS3 (an important component of the RNA silencing pathway) [74]. Thus, potyviruses have also evolved like animal viruses in combating the host defense mechanism and promoting their infection [75]. All these findings thus highlight the multifunctional roles of NIb which is attributed to the interaction network of NIb involving both the viral as well as host proteins thereby playing a crucial role in potyviral infection cycle.

## 5. Regulation of the Potyviral Proteins during Various Events in the Viral Life Cycle

All the potyviral proteins are multifunctional and have more than one interacting partner protein and participate in various processes of the viral life-cycle that need to be regulated. The regulation can be brought about by the post-translational modifications of the proteins, regulation of the production of proteins, interaction with various host factors, etc.

Since all potyviral proteins are generated in equimolar amounts (except P3N-PIPO and P1N-PISPO, formed by transcriptional slippage), some of the proteins are synthesized more than the catalytic levels required for infection. Compartmentalization of proteins into inclusion bodies in the nucleus (NIa, NIb, P3) and within the cytoplasm (CI protein) is one of the methods of reducing the cytoplasmic concentrations of the excess proteins [65,76]. In fact, this is how NIa, NIb and CI have derived their names. PVBV VPg has a bipartite nuclear localization signal (NLS) at its N-terminus (NLS I: 6–14, NLS II: 44–52; PVBV numbering) which targets VPg-Pro to the nucleus and regulates the concentration of VPg-Pro present in the cytoplasm at any given time [65,77]. NLS I has been shown to be essential for localization of VPg-Pro in Cajal bodies, while Lys 42 and Lys 44 of the NLS II are crucial for localization to nucleolus within the plant cell nuclei [54]. It was demonstrated that the nuclear fractions of PVBV-infected plant cells contained VPg-Pro which was capable of Mg^2+^ -dependent DNase activity, by virtue of the protease domain involving the Asp 81 as an essential residue required for metal ion-binding. It was proposed that DNase activity of VPg-Pro was responsible for host genomic DNA degradation. The VPg-Pro gets accumulated in the nucleus during the late stage of potyviral infection, i.e., upon completion of viral RNA synthesis. Therefore, VPg-Pro may be capable of suppressing silencing in the nucleus [54,77]. Additionally, the infected plant nuclear extract containing VPg-Pro was also demonstrated to have the protease activity [77].

The suboptimal cleavage between VPg and NIa-Pro is also another means of regulation. Similarly, appearance of other stable polyprotein intermediates has also been reported for example, 6 K2-VPg-Pro, which could be directed to membranes due to the hydrophobic domain of 6K2 [39], P3-6K1 [78] and others. Thus, temporal regulation of polyprotein processing is crucial for a successful virus life cycle. The VPg-mediated modulation of the protease activity of NIa-Pro also forms another layer of regulation during a particular stage of the viral life cycle [24]. VPg and NIa-Pro, both can undergo post-translational modifications and get phosphorylated by host kinases. The host cell kinases could phosphorylate the lone NIa-Pro domain, thereby reducing its protease activity and preventing infection. However, when these two domains are present together, the phosphorylation site is masked. Therefore, a suboptimal cleavage between VPg and NIa-Pro could prevent the phosphorylation of NIa-Pro which would thus prevent such inhibition by the host [50]. The presence of an autoinhibitory motif at the N-terminus of VPg could also be responsible for regulating the activity of VPg-Pro [28].

Regulating the production of protein levels at different stages of viral life cycle is another way of regulation. This is achieved by controlling the turnover number of the proteins. For instance, high levels of CP, generated upon efficient cleavage from NIb-CP precursor, have been shown to inhibit replication-associated potyviral gene expression. To avoid premature encapsidation of the viral genome, concentration of the CP needs to be regulated. It has been proposed that the CP levels are regulated by a co-chaperone that delivers CP to Hsp70, namely CPIP (coat-protein interacting protein), which promotes ubiquitination and degradation of CP. In the later stages of infection, when replication has been completed, CPIP gets depleted and CP is made available for viral assembly [79]. Similarly, CP also inhibits the potyviral RNA translation during the late stages of the infection so that more RNA is available for encapsidation and thus facilitating the assembly of the virus [80]. Potyviral proteins are also subjected to post-translation modifications such as phosphorylation (VPg, NIa-Pro and CP), glycosylation (CP) and SUMOylation (NIb) [33,36,81,82,83] which forms another line of regulation during the viral life-cycle.

## 6. Applications of Potyviruses in Biotechnology: Characterization of PVBV VLPs as Nanoparticles and Applications of PVBV Chimeric VLPs

Potyviruses and their encoded proteins are used in various biotechnological applications. Different potyviruses are used as vectors for the expression of heterologous proteins in plants. There are insertion sites in potyvirus at the 5′ end of the P1 cistron [84], between P1 and HC-Pro [85] and between the NIb and CP cistrons [86] where various genes can be inserted for their expression. These sites are then proteolytically processed by the viral-encoded proteases, thereby releasing the protein of interest. This property of the potyviruses is therefore exploited for the heterologous expression of proteins. Apart from the virus itself, the potyviral proteins also have a number of other applications. Owing to the high specificity and affinity of NIa-Pro, it is used to remove tags which are added for increasing the solubility and yield of recombinant proteins [87,88,89,90]. As mentioned earlier, the CP can self-assemble to form VLPs, therefore these VLPs have been exploited for use as a nanocarrier for a large number of biomedical applications. A large number of plant VLPs/plant virus nanoparticles (PVNs) are used in various biomedical applications such as vaccination, imaging, gene delivery [91]. The filamentous rod-shaped viruses have a large surface area to volume ratio, thus providing a high payload capacity and giving them an edge over the icosahedral viruses. There are a few potyviruses which have been studied for various applications in nanotechnology. For instance, PPV and TuMV have been used for antigen presentation and thus a possible application in vaccine [92,93]. The potyvirus-based nanoparticles have also been used as enzyme nano-carriers (ENCs) where the enzymes of the resveratrol synthetic pathway were immobilized on PVA particles. These immobilized enzymes were thus able to synthesize resveratrol [94]. To explore the possibility of PVBV VLPs as nanocarriers, the modes of internalization of PVBV VLPS in different mammalian cells, their intracellular fate and endocytic uptake pathways were studied [95]. It was proposed that a clear understanding of nanoparticle-cell interactions is important for developing efficient targeted delivery systems.

Vimentin and Hsp60 were identified as the surface-expressed proteins which facilitated the internalization of PVBV VLPs in HeLa and HepG2 cells, respectively. The major mode of internalization of PVBV VLPs was shown to be caveolae-mediated endocytosis in both HeLa and HepG2 cells. The caveolae-mediated endocytosis is of great importance in nanomedicine since it can bypass the lysosomes, although with few exceptions. Furthermore, this pathway could also be utilized for transvascular delivery of nanomaterials as it is a distinguished transendothelial pathway [96]. The animal picornaviruses such as enterovirus 71 and Theiler’s murine encephalomyelitis virus (TMEV) also internalize into infected cells via vimentin [97,98]. Thus, potyvirus VLPs use the same attachment/entry mechanisms into mammalian cells as animal picornaviruses [99]. It was proposed that PVBV VLPs traffic through endolysosomal pathway after internalization to finally get degraded in the lysosomes. Since PVBV VLPs traffic via endolysosomal pathway, we speculate that this could pave the way for studies on the role of endocytosis in potyvirus infection which remains elusive till date. In the case of TuMV, a plant dynamin-related protein 1 (DRP1, a GTPase involved in endocytosis) from *Arabidopsis thaliana* (AtDRP1A) was shown to promote TuMV infection. AtDRP1A interacts with VPg and CI, which are both an important component of viral replication complex (VRC). In TuMV-infected cells, it was observed that AtDRP1A also colocalized with VRC. An adaptor protein AP2 was also shown to be an important host factor for TuMV infection [100]. Additionally, it was also observed that an inhibition of dynamin not only disrupted endocytosis of TuMV, but also inhibited virus replication and intercellular movement [101]. Taken together, these findings thus shed light on the role of components of the endocytic pathway in potyvirus infection.

**Figure 3:**
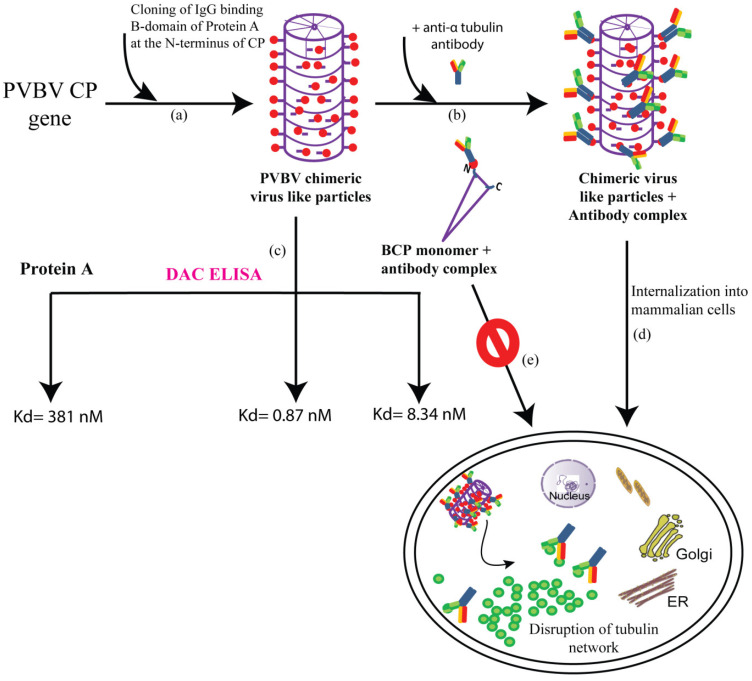
Schematic representation of the generation of chimeric VLPs and their potential applications (**a**) IgG-binding B-domain is cloned at the N-terminus of CP gene which assembles to form PVBV chimeric VLPs with each subunit expressing the B-domain at its N-terminus (**b**) Incubating the PVBV chimeric VLPs with anti-α tubulin antibody led to the formation of chimeric VLPs + antibody complex (**c**) PVBV chimeric VLPs, protein A as well as the monomer BCP (PVBV CP N-terminal fusion with B-domain) subunit + antibody complex were subjected to DAC ELISA (direct antigen coating-enzyme linked immunosorbent assay) where an antibody unrelated to CP was used. The dissociation constant (Kd) for each of the complexes is depicted, indicating an approximately 500-fold higher antibody-binding affinity of chimeric VLPs when compared to protein A (**d**) chimeric VLPs + anti-α tubulin antibody complex could internalize into mammalian cells and deliver the functional tubulin antibodies, which caused the disruption of the tubulin network ultimately causing cell death (**e**) whereas the BCP monomer (Ni-NTA purified BCP after size exclusion chromatography) + antibody complex fail to enter the mammalian cells.

One of the major challenges in immunotherapy, is that the antibodies do not have the ability to enter cells. Therefore, clinical applications of antibodies are limited to those against surface exposed antigens [102]. Thus, antibodies to intracellular antigens specific to disease as therapeutics are largely unexplored. With a view to develop PVBV VLP-based nanocarriers for the delivery of antibodies, the B domain of *Staphylococcus aureus* protein A (SpA) (that binds to the constant region of the antibodies) was genetically engineered at the N-terminus of PVBV CP [103]. PVBV CP N-terminal fusion was designated as BCP and assembled virus particles as chimeric PVBV VLPs. The chimeric VLPs generated were shown to have ~500-fold higher IgG-binding affinity than SpA. Such PVBV chimeric VLPs could be of use for developing more sensitive immunodiagnostics. The chimeric PVBV VLPs, like the PVBV VLPs could internalize into various mammalian cells and were degraded after 10 h. The high affinity of the chimeric PVBV VLPs towards antibodies enabled easy generation of chimeric VLP-antibody complexes (for example, anti-CD20 and anti-α tubulin) which have been shown to successfully deliver the bound antibodies intracellularly. Further, the delivered antibodies were also functional inside the cells. Therefore, this property of chimeric PVBV VLPs to bind and deliver antibodies intracellularly could pave the way for future applications in therapeutics [104]. Interestingly, only the assembled VLPs could internalize into the cells. BCP purified by Ni-NTA chromatography formed a monomer as per size exclusion chromatography and hence termed as BCP monomer. This BCP monomer failed to interact with vimentin and thus could not internalize into the cells. Thus, it is the assembled scaffold of the viruses that recognize the surface receptors on the host cells and gain entry. The requirement of assembled VLPs also highlights the importance of CP subunits interaction in the development of potyvirus infection. The generation of PVBV chimeric VLPs and their future applications are depicted in Figure 3.

## 7. Conclusions and Future Prospects

*Potyvirus* biology has been extensively studied and advanced considerably during the last decade. With the increasing evidence of the functions and interacting partners of various potyvirus-encoded proteins, it is now being understood that most of these potyviral proteins are multifunctional playing important roles in more than one viral process. Therefore, the functional regulation of these proteins, by different means such as protein–protein interactions, post-translational modifications, forms an interesting avenue for research that could provide further insights into the complexity of potyviral life cycle.

Understanding the process of transmission of potyviruses, although challenging could open new avenues to control the vector populations as well as dissemination of the potyviral infection in future. Deciphering of viral receptors in the insects is a prerequisite to understand the transmission process. The various animal picornavirus-receptor interactions could be helpful in achieving the same by looking for their counterparts in insects. Internalization of PVBV VLPs via surface-expressed vimentin could also provide some impetus to explore this membrane protein in insects for interaction with potyvirus proteins. While the components of endocytosis during potyviral infection in plant cells is beginning to be identified now, identifying the host proteins that participate in endocytic pathways could also help to control the potyviral infection. As mentioned earlier, AtDRP1A, a host factor involved in TuMV infection could also be a potential target in future for generating potyvirus-resistant plants. Potyviruses exhibit replication-associated translation (RAT) [58], however, the coordination between these two steps still remains unanswered. Even the mechanism of switching of VRCs from synthesizing negative-strand viral RNA to the plus-strand RNA needs to be elucidated [104].

Although, VPg-Pro has been extensively studied in different potyviruses and is also discussed in this review, still much more remains unexplored. The requirement of ATPase activity of VPg-Pro during different events such as intracellular movement, replication, translation and polyprotein processing, host RNA silencing needs to be understood. This would give mechanistic insights into the potyviral infection. Since ∆N22 VPg has been shown to be a functional globular protein, its crystallization and structure determination can be attempted. Since VPg-Pro has ATPase function, it would also be interesting to examine if VPg-Pro exhibits helicase activity. The motifs and residues involved in the function and regulation of PVBV NIa-Pro and VPg has now been established in a heterologous system. Therefore, it would be interesting to understand if tampering with such motifs/residues would affect PVBV infection.

Flexuous rods are difficult to crystallize, and the cryo EM structure of only two potyviruses, viz watermelon mosaic virus and PVY have been determined so far at 4 Å and 3.4 Å resolution, respectively [105,106]. Attempts can also be made to crystallize the octameric 16 S ring which is devoid of the N- and C-terminal residues of CP. It would also be interesting to understand how these IDPRs in CP modulate the function of its cognate partner-binding proteins. Both CP and VPg interact with each other and also are involved in the cell-to-cell/long distance movement of the virus, therefore identifying the domain of interaction between them would open new avenues for research in terms of the regulation of the systemic infection of the virus.

The chimeric VLPs of potyviruses hold promise for various biomedical applications. These need to be explored further by in vivo studies.
